# HSI2/VAL1 and HSL1/VAL2 function redundantly to repress *DOG1* expression in Arabidopsis seeds and seedlings

**DOI:** 10.1111/nph.16559

**Published:** 2020-04-25

**Authors:** Naichong Chen, Hui Wang, Haggag Abdelmageed, Vijaykumar Veerappan, Million Tadege, Randy D. Allen

**Affiliations:** ^1^ Institute for Agricultural Biosciences Oklahoma State University Ardmore OK 73401 USA; ^2^ Department of Biochemistry and Molecular Biology Oklahoma State University Stillwater 74078 OK USA; ^3^ Department of Agricultural Botany Faculty of Agriculture Cairo University Giza 12613 Egypt; ^4^ Department of Biology Eastern Connecticut State University Willimantic CT 06226 USA; ^5^ Department of Plant and Soil Sciences Oklahoma State University Stillwater OK USA

**Keywords:** dormancy, germination, histone methylation, polycomb repressive complex 2, transcriptional repression

## Abstract

- *DELAY OF GERMINATION1* (*DOG1*) is a primary regulator of seed dormancy. Accumulation of DOG1 in seeds leads to deep dormancy and delayed germination in Arabidopsis. B3 domain‐containing transcriptional repressors HSI2/VAL1 and HSL1/VAL2 silence seed dormancy and enable the subsequent germination and seedling growth. However, the roles of HSI2 and HSL1 in regulation of *DOG1* expression and seed dormancy remain elusive.
- Seed dormancy was analysed by measurement of maximum germination percentage of freshly harvested Arabidopsis seeds. *In vivo* protein–protein interaction analysis, ChIP‐qPCR and EMSA were performed and suggested that HSI2 and HSL1 can form dimers to directly regulate *DOG1*.
- HSI2 and HSL1 dimers interact with RY elements at *DOG1* promoter. Both B3 and PHD‐like domains are required for enrichment of HSI2 and HSL1 at the *DOG1* promoter. HSI2 and HSL1 recruit components of polycomb‐group proteins, including CURLY LEAF (CLF) and LIKE HETERCHROMATIN PROTEIN 1 (LHP1), for consequent deposition of H3K27me3 marks, leading to repression of *DOG1* expression.
- Our findings suggest that HSI2‐ and HSL1‐dependent histone methylation plays critical roles in regulation of seed dormancy during seed germination and early seedling growth.

## Introduction

Seed dormancy is an adaptive mechanism that allows for the dispersal and survival of seeds over distance and time, and ensures that germination occurs under favourable conditions (Finch‐Savage & Leubner‐Metzger, 2006). In agricultural settings, seed dormancy can affect the competitiveness of weeds during crop production, so understanding the genetic, molecular, physiological and environmental factors that affect seed germination under field conditions could lead to new, more environmentally benign, weed control strategies that could improve agricultural sustainability (Finch‐Savage & Footitt, 2017). Seed dormancy is a complex trait that is regulated by both phytohormones and genetic factors. Abscisic acid (ABA) plays an important role in initiating and enhancing seed dormancy, while the dormant state is reversed by gibberellins, which promote germination under favourable conditions (Koornneef *et al.*, 2002; Liu *et al.*, 2010). Recently, *DELAY OF GERMINATION1* (*DOG1*) was reported to be a major quantitative trait locus for the genetic regulation of seed dormancy in Arabidopsis (Alonso‐Blanco *et al.*, 2003; Bentsink *et al.*, 2006, 2010; Huang *et al.*, 2010). Compared with wild‐type, loss‐of‐function *dog1‐3* Arabidopsis mutant seeds germinated early, whereas gain‐of‐function *dog1‐5* mutant seeds showed delayed germination (Cyrek *et al.*, 2016; Huo *et al.*, 2016). DOG1 acts in parallel with ABA to delay germination (Graeber *et al.*, 2014) and, since DOG1 requires the clade A PP2C phosphatases PP2C to control seed dormancy (Née *et al.*, 2017), the ABA and DOG1 pathways converge at that level. In addition, ETHYLENE RESPONSE FACTOR12 (ERF12), and its downstream target ETHYLENE RESPONSE1 (ETR1), directly bind the *DOG1* promoter and recruit TOPLESS (TPL), leading to repression of *DOG1*, suggesting that ethylene also regulates seed dormancy through the ETR1–ERF12/TPL–DOG1 module (Li *et al.*, 2019). Low temperature during seed maturation increases *DOG1* expression and induces seed dormancy (Chiang *et al.*, 2011; Kendall *et al.*, 2011; Nakabayashi *et al.*, 2012). Seed development under low‐temperature conditions triggers *DOG1* expression by increasing the expression and protein abundance of bZIP67, which directly targets the *DOG1* promoter and activates *DOG1* expression, leading to enhanced seed dormancy (Bryant *et al.*, 2019). *DOG1* is also regulated by other complex mechanisms that include histone modifications, alternative polyadenylation, alternative splicing, and a *cis*‐acting antisense noncoding transcript (*asDOG1*) (Bentsink *et al.*, 2006; Müller *et al.*, 2012; Molitor *et al.*, 2014; Cyrek *et al.*, 2016; Fedak *et al.*, 2016). In addition to seed dormancy, high‐level expression of *DOG1* leads to increased drought tolerance in Arabidopsis (Yatusevich *et al.*, 2017), and DOG1 was also found to regulate flowering in lettuce and Arabidopsis through a microRNA‐mediated pathway (Huo *et al*, 2016).

Two closely related proteins, HIGH‐LEVEL EXPRESSION OF SUGAR INDUCIBLE2 (HSI2) and HSI2‐LIKE1 (HSL1), play critical roles in the developmental transition from seed maturation to germination and seedling development and from vegetative growth to reproductive development (Tsukagoshi *et al.*, 2005, 2007; Suzuki *et al.*, 2007; Veerappan *et al.*, 2012; Chhun *et al.*, 2016; Qüesta *et al.*, 2016; Yuan *et al*., 2016; Chen *et al.*, 2018). These proteins, also known as VIVIPAROUS‐1/ABSCISIC ACID INSENSITIVE 3‐LIKE1 (VAL1) and VIVIPAROUS‐1/ABSCISIC ACID INSENSITIVE 3‐LIKE2 (VAL2), contain four conserved putative functional domains, including a plant homeodomain (PHD)‐like, a plant‐specific B3 DNA binding domain, a cysteine‐ and tryptophan‐rich zinc finger domain (CW), and an ethylene‐responsive element binding factor‐associated amphiphilic repression (EAR) domain. The HSI2 B3 domain binds to RY/Sph (RY) elements and is required for HSI2‐dependent repression of *FLOWERING LOCUS C* (*FLC*) and *AGAMOUS‐LIKE15* (*AGL15*) (Qüesta *et al.*, 2016; Yuan *et al*., 2016; Chen *et al.*, 2018). The HSI2 PHD domain is also required for HSI2 accumulation at the *AGL15* locus and repression of *AGL15* expression (Chen *et al.*, 2018). HSI2 and HSL1 can form homodimers and heterodimer *in vivo* and HSL1 shows partial redundancy to HSI2 in the regulation of *FLC* (Chhun *et al.*, 2016; Yuan *et al*., 2016). HSI2 is reported to recruit polycomb repressive complex 2 (PRC2) to the *AGL15* and *FLC* loci (Qüesta *et al.*, 2016; Yuan *et al*., 2016; Chen *et al.*, 2018). HSI2 was also reported to bind HISTONE DEACETYLASE6 (HDA6) and MEDIATOR13 (MED13), while HSL1 was reported to interact with HDA19, indicating that these factors may also be involved in the repression of seed maturation genes during germination in Arabidopsis (Zhou *et al.*, 2013; Chhun *et al.*, 2016). The molecular mechanisms of HSL1 function and its relationship with HSI2 have not been fully characterised.

LIKE HETEROCHROMATIN PROTEIN1 (LHP1) recognises H3K27me3 (trimethylation of lysine at histone H3) repressive marks through its chromodomain (Turck *et al.*, 2007; Zhang *et al.*, 2007; Exner *et al.*, 2009), and interacts with MULTICOPY SUPPRESSOR OF IRA1 (MSI1) to positively recruit PRC2 to chromatin for the deposition of H3K27me3 (Derkacheva *et al.*, 2013). LHP1 also directly interacts with RING‐RAWUL proteins and EMBRYONIC FLOWER1 (EMF1, a component of PRC1), suggesting that LHP1 can be present in several PRC1‐like complexes and may act as a bridge between PRC1 and PRC2 (Xu & Shen, 2008; Bratzel *et al.*, 2010). In Arabidopsis, LHP1 co‐localises with H3K27me3 across the genome, and is responsible for the expansion of H3K27me3 associated with the stabilisation of transcriptional repression (Turck *et al.*, 2007; Zhang *et al.*, 2007; Exner *et al.*, 2009). Expression of many tissue specific genes is upregulated in *lhp1* loss‐of‐function mutants (Libault *et al.*, 2005; Lafos *et al.*, 2011), with decreased levels of H3K27me3 seen at direct target gene loci, including *FLC* (Veluchamy *et al.*, 2016; Yuan *et al*., 2016), indicating that LHP1‐dependent gene repression correlates with deposition of H3K27me3. Molitor *et al. *(2014) reported that *DOG1* is negatively regulated by ALFIN1‐like proteins, which contain PHD domains and can form protein complexes with LHP1 at the *DOG1* locus to replace H3K4me3 (trimethylation of lysine 4 at histone H3) marks associated with gene activation with H3K27me3, leading to its transcriptional downregulation and promotion of seed germination.

Here we report that the proximal region of the *DOG1* promoter is directly targeted by both HSI2 and HSL1. These transcriptional repressors recruit LHP1 and CURLY LEAF (CLF), and promote the deposition of H3K27me3 marks, leading to repression of *DOG1*. Delayed germination in loss‐of‐function *hsi2 hsl1* mutants indicates that HSI2‐dependent silencing of *DOG1* promotes the early release of seed dormancy.

## Materials and Methods

### Plant materials and growth conditions

Arabidopsis (*Arabidopsis thaliana*) Columbia (Col‐0; CS60000) wild‐type, loss‐of‐function alleles *hsl1‐1* (SALK_059568), *lhp1‐1* (CS3796) and *dog1‐3* (SALK_000867), and gain‐of‐function allele *dog1‐5* (SALK_022748) were obtained from Arabidopsis Biological Resources Center. For all the experiments, plants were grown under continuous illumination (fluorescent lamps at *c.* 200 µmol m^−2^ s^−2^) at 24°C on 0.3% Phytagel plates containing ½ Murashige and Skoog (MS) salt, 0.5 g l^−1^ MES, 1× Gamborg vitamin mix, and 1% sucrose (pH adjusted to 5.7).

### Germination assays

Freshly harvested seeds were plated on Petri dishes (100 seeds per dish) containing 0.5% Phytagel and incubated under long‐day conditions (16 h : 8 h, light : dark, 25°C : 20°C cycle). Radicle emergence was scored after 3 d and the percentage of total seeds germinated was calculated. Data presented are the means ± SD of three independent assays with seeds from different plants.

### Plasmid construct and plant transformation

The *HSL1* promoter, consisting of a 3687‐bp fragment immediately upstream of the HSL1 start codon, was amplified by PCR and cloned into the binary vector pDONR207. After sequencing, the *HSL1* promoter was cleaved by *Apa*I restriction enzyme and inserted into pGWB504 containing *HSL1* cDNA to generate the *HSL1pro:HSL1* DNA construct that contains a green fluorescent protein (GFP) tag at the C terminus. To generate the *35Spro:LHP1* construct, pDONR207 containing the full‐length *LHP1* coding sequence was subcloned into the destination vectors pGWB521 and pEarleygate104. Arabidopsis plants were transformed using the floral dip method (Clough & Bent, 1998). The primers used for generating these constructs are listed in Supporting Information Dataset S1.

### Luciferase imaging

Imaging of luciferase was performed using an Andor iKON‐M DU934N‐BV charge‐coupled device (CCD) camera (Andor Technology, Belfast, UK). Andor Solis (I) imaging software (Andor Technology) was used for image acquisition and processing. Before luminescence imaging, Arabidopsis siliques and infiltrated *Nicotiana benthamiana* leaves were uniformly sprayed with 5 mM d‐luciferin (Gold Biotechnology, Olivette, MO, USA) in 0.01% Triton X‐100 solution. After spraying with luciferin, silique and leaf samples were incubated in the dark for 5 min. Exposure time for luminescence imaging was 10 min, unless otherwise specified.

### Transient expression assays

A 1.5 kb DNA sequence immediately upstream of the translational start codon of *DOG1* was cloned into the pENTR/D‐TOPO vector, and then the *DOG1* promoter sequence and mutant derivatives of *DOG1* promoter (mRY1, mRY2 and mRY1/2) were subcloned into pGWB535 using the Gateway system to generate reporter constructs. The reporter constructs and effector constructs that express 35Spro:HSI2, 35Spro:HSI2mB3, 35Spro:HSI2mPHD, 35Spro:HSI2mCW and 35Spro:HSI2mEAR were introduced into *Agrobacterium tumefaciens* (GV2260) for subsequent agroinfiltration. After 2 d of growth, the infiltrated leaves were sprayed with luciferin and imaged by CCD camera. The infiltrated regions were excised and total RNA was extracted. Each experiment was performed with at least three replicates. For transient expression in protoplasts, the effector construct that expresses HSI2pro:HSI2‐GFP (Chen *et al.*, 2018) was cotransformed with reporter constructs into Arabidopsis protoplasts. Transient expression assays were performed with three replicates using Arabidopsis protoplasts as described (Asai *et al.*, 2002; Zhang *et al.*, 2014). For each transformation, 5 µg of reporter and 4 µg of effector plasmid were used. For normalisation of the activity of the reporter gene, 1 µg of plasmid pRLC was used as an internal control.

### Reverse transcriptase quantitative PCR assays

Reverse transcriptase quantitative PCR (RT‐qPCR) assays was performed using a StepOne Plus system (Applied Biosystems, Waltham, MA, USA) with iTaqSYBR Green Supermix with 6‐carboxy‐X‐rhodamine (Bio‐Rad, Hercules, CA, USA). RNase‐free DNase (Qiagen)‐treated total RNA was used for cDNA synthesis using the iScript cDNA synthesis kit (Bio‐Rad). For each experiment, cDNA synthesis reactions were performed on three independent RNA samples prepared from *c.* five seedlings, each. Three qPCR reactions were performed for each cDNA sample. *EF1A* (AT5G60390) and *HYGROMYCIN PHOSPHOTRANSFERASE* (*HPT*) were used as reference genes for Arabidopsis and *N. benthamiana*, respectively. The relative expression of genes was calculated according to the ABI Prism 7700 sequence detection system (User Bulletin no. 2). Primer sequences used for RT‐qPCR are listed in Dataset S1.

### Chromatin immunoprecipitation‐qPCR analysis

Chromatin immunoprecipitation‐qPCR (ChIP) assays were performed as previously described (Chen *et al.*, 2018). Due to technical difficulties encountered with ChIP assays in mature seeds, chromatin was extracted from 7‐d‐old seedlings grown in MS medium supplemented with 1% sucrose. The chromatin in these seedlings was cross‐linked with 1% formaldehyde. The resulting chromatin was sheared to fragments with a 500‐bp (200–1000‐bp) average length by sonication and used for immunoprecipitation with commercially available anti‐GFP (Abcam, ab290), anti‐HA (Abcam, ab9110) and anti‐H3K27me3 (Millipore, 07‐449), respectively. After reversing the cross‐links, immunoprecipitated DNA was analysed by qPCR using primers for specific regions of the *DOG1* gene. Three independent experiments, each using 500 mg of seedlings (25–30 individual plants), were performed. Three technical replicates for each qPCR assay were performed and data from each individual experiment were calibrated by input, and normalised using *ACT2*. In Arabidopsis leaf protoplasts, the ChIP assays were performed as described previously (Lee *et al.*, 2007; Du *et al.*, 2009; Xiong *et al.*, 2013; Zhang *et al.*, 2014). *HSL1pro:HSL1‐GFP* DNA was transformed into *hsl1‐1* and *hsi2‐2* Arabidopsis protoplasts from 14‐d‐old leaves using the polyethylene glycol‐mediated transformation method. Protoplasts were incubated at room temperature for 13 h under dark conditions. Protoplast chromatin was cross‐linked by 1% formaldehyde in W5 medium for 20 min and quenched with glycine (0.2 M) for 5 min. The protoplasts were then lysed, and the DNA was sheared on ice with sonication. Immunoprecipitation was performed with anti‐GFP. After reversing the cross‐links, the purified DNA was analysed by qPCR using primers for specific regions of *DOG1*. Each experiment was repeated at least twice using protoplasts from *c.* five leaves. Three technical replicates for each qPCR assay were performed, and *ACT2* was used as an internal control for normalisation. Primers of target genes used for qPCR in ChIP analysis are listed in Dataset S1.

### Electrophoretic mobility shift assays

Electrophoretic mobility shift assays (EMSA) were performed using biotinylated double‐stranded DNA probes and the Lightshift Chemiluminescent EMSA kit (Thermo Scientific, Waltham, MA, USA). Biotin 3ʹ end‐labelled DNA oligomers were prepared using a biotin end‐labelling kit (Thermo Scientific), and double‐stranded DNA probes were generated by annealing sense and antisense oligomers. Each 20‐ml binding reaction contained 50 fmol of biotin‐labelled double‐stranded DNAs, 5 mg of recombinant His‐HSI2B3 protein, and 50 ng ml^−1^ poly(dIdC) in binding buffer (10 mM Tris, 30 mM KCl, 0.1 mM EDTA, 1 mM dithiothreitol (DTT), 0.05% Nonidet P‐40 and 6.5% glycerol, pH 7.9). Binding reactions were incubated for 20 min at room temperature and resolved by electrophoresis of reaction samples on 5% polyacrylamide gels with TBE buffer. Detection of biotin‐labelled DNA was performed using an Andor iKON‐M DU934N‐BV CCD camera (Andor Technology).

### Yeast‐two‐hybrid analysis

Yeast‐two‐hybrid assays were performed using the Matchmaker GAL4‐based two‐hybrid system 3 (Clontech) according to the manufacturer's instructions. Sequences that encode full‐length HSI2, PHD_HSI2_ and HSI2mPHD were subcloned into the pGADT7 and pGBKT7 vectors, whereas the full‐length HSL1 coding sequences were subcloned into the pGBKT7 vector. All constructs were transformed into yeast strain AH109 using the lithium acetate method, and yeast cells were grown on a minimal medium/−Leu/−Trp according to the manufacturer’s instructions (Clontech). Transformed colonies were plated onto a minimal medium/−Leu/−Trp/−His/−Ade to test for possible interactions.

### Bimolecular florescence complementation analysis

For bimolecular florescence complementation (BiFC) assays, full‐length *LHP1* cDNA was cloned into the pDONOR207 vector and subsequently introduced into the pSITE‐DEST‐nEYFP‐C1 vector containing the N‐terminal fragment of yellow fluorescent protein (YFP). The coding sequences of *HSL1*, *HSI2*, *HSI2mB3*, and *HSI2mPHD* were initially cloned into the pENTR/SD/D‐TOPO vector and subcloned into pSITE‐DEST‐nEYFP‐C1 and pSITE‐DEST‐cEYFP‐C1 vectors. *Agrobacterium tumefaciens* strain GV2260 carrying each construct was co‐infiltrated into tobacco leaves. After 2 d, YFP fluorescence signal was observed using a laser scanning confocal microscope (Leica TCS SP8 confocal) at the Noble Research Institute Imaging Core Facility.

### *In vivo* co‐immunoprecipitation assay

For co‐immunoprecipitation (Co‐IP) assays, total proteins from homogenised tobacco were extracted using extraction buffer (50 mM Tris‐HCl, pH 7.5, 150 mM NaCl, 2 mM EDTA, 10% glycerol, 0.2% 2‐mercaptoethanol, 1% Triton X‐100, 1 mM phenylmethylsulfonyl fluoride (PMSF), and 1× protease inhibitor cocktail) and incubated for 30 min at 4°C with gentle agitation. After centrifugation at 12 000 ***g*** for 15 min at 4°C, supernatant was incubated with protein A + G magnetic beads (Millipore 16‐663) for 1 h for preclearing at 4°C. The precleared protein solution was incubated with 50 µl anti‐GFP mAb‐magnetic beads (MBL D153‐11) overnight at 4°C. The supernatant was removed, and the magnetic beads were washed three times with extraction buffer. The proteins were eluted with 2× SDS sample loading buffer and analysed by immunoblotting using anti‐*myc* antibody (MA1‐980; Thermo Fisher, Waltham, MA, USA).

## Results

### HSI2 and HSL1 redundantly repress *DOG1* and affect seed dormancy

**Fig. 1:**
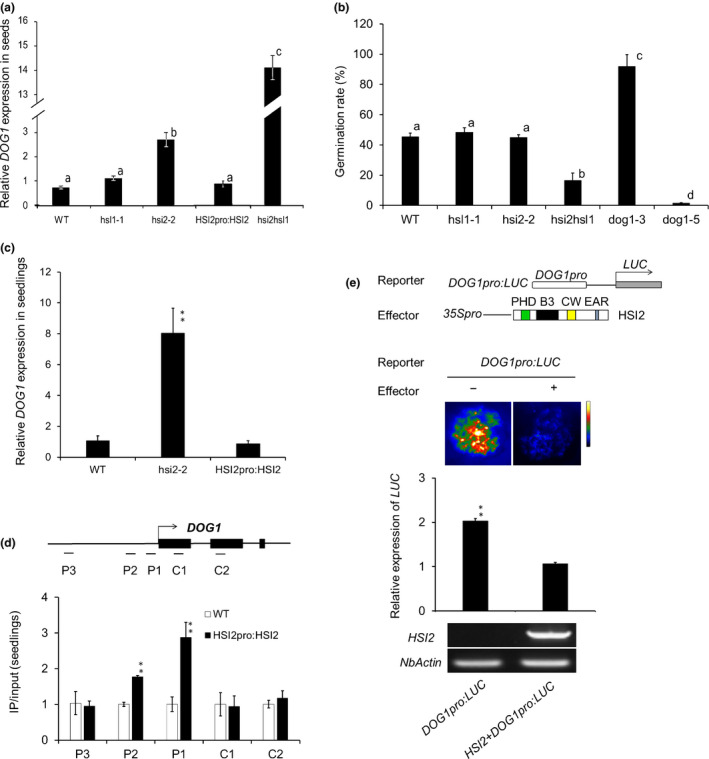
*hsi2 hsl1* double mutant shows increased primary seed dormancy and HSI2 directly binds to *DOG1* to regulate its expression. (a) Relative expression of *DOG1* in freshly harvested seeds from wild‐type (WT), *hsl1‐1*, *hsi2‐2*, *HSI2pro:HSI2* and *hsi2 hsl1* Arabidopsis plants. RT‐qPCR assays were normalised using *EF1a*. (b) Maximum germination percentage of freshly harvested seeds of *hsl1‐1*, *hsi2‐2* and *hsi2 hsl1*. Seeds from *dog1‐3* loss‐of‐function and *dog1‐5* gain‐of‐function mutants were used as controls. Rates of germination were scored 3 d after plating. Means of three independent experiments (±SD) are shown. Lowercase letters indicate significant differences (*P* < 0.01) between genotypes. (c) Relative expression of *DOG1* in WT, *HSI2pro:HSI2* and *hsi2‐2* Arabidopsis seedlings, assayed by RT‐qPCR. (d) ChIP‐qPCR analysis of HSI2 enrichment at *DOG1* gene in WT and *HSI2pro:HSI2* Arabidopsis seedlings. Schematic representations of *DOG1* gene and assayed genomic regions are indicated, with P indicating promoter regions and C indicating coding regions. These regions were analysed by ChIP‐qPCR analysis of 7‐d‐old transgenic seedlings harbouring *HSI2pro:HSI2‐HA* using HA antibody. qPCR data was normalised using *ACT2* as an internal standard. Data represent the means of three qPCR reactions from each of three independent ChIP assays. (e) Schematic representation of reporter and effector used to assay the function of the HSI2 in the regulation of *DOG1*. The reporter construct includes a luciferase gene is driven by the *DOG1* promoter. In the effector construct, expression of HSI2 is controlled by the *CaMV 35S* promoter. Luminescence images and relative expression of luciferase mRNA, determined by RT‐qPCR assays, in *Nicotiana benthamiana* leaves co‐infiltrated with the reporter and effector gene combinations as indicated. Expression of the *NbActin* gene was used for normalisation. Data represent the means of three qPCR reactions from each of three independent assays. Error bars in indicate SD. **Indicate statistically significant differences (*P* < 0.01) determined by Student’s *t*‐test.

Reverse transcription quantitative PCR (RT‐qPCR) analysis showed that *DOG1* expression in freshly harvested *hsi2‐2* Arabidopsis seeds increased by three‐fold, relative to wild‐type (WT), while expression in *hsl1‐1* seeds or seeds from *hsi2‐2* plants complemented with *HSI2pro:HSI2* was not significantly affected (Fig. 1a). Conversely, a 14‐fold increase in *DOG1* expression was detected in *hsi2 hsl1* double mutant seeds. Analysis of the per cent germination of freshly harvested seeds from these Arabidopsis lines confirmed that loss‐of‐function mutations in both *hsi2* and *hsl1* are necessary for significant increases in seed dormancy to be detected (Fig. 1b). The gain‐of‐function mutant *dog1‐5*, which shows strongly delayed germination, and the loss‐of‐function mutant *dog1‐3*, which shows early germination, served as controls in this assay. These results indicated that HSI2 and HSL1 act redundantly to repress *DOG1* expression in seeds. Although their functions overlap, the native *HSI2* gene is able to fully complement *HSL1*, while *HSL1* cannot fully replace the function of *HSI2*. Furthermore, while seed dormancy is promoted by *DOG1* expression, it appears that a relatively large increase in *DOG1* expression, as seen in the *hsi2 hsl1* double mutant, is necessary to significantly increase seed dormancy.

### HSI2 directly regulates *DOG1* by binding to its promoter

As expression of *DOG1* is upregulated and H3K27me3 enrichment at the *DOG1* locus is reduced in *hsi2‐2* knockout mutant Arabidopsis seedlings (Veerappan *et al.*, 2012, 2014), we predicted that *DOG1* could be a direct regulatory target of HSI2. To test this hypothesis, a rescued *hsi2‐2* Arabidopsis line that expresses HA epitope‐tagged HSI2 under the control of the native *HSI2* promoter (*HSI2pro:HSI2‐HA*) was developed. Repression of *DOG1* was restored in this line with expression reduced, relative to *hsi2‐2*, to levels similar to WT seedlings (Fig. 1c). To detect and quantify the enrichment of HSI2 at the *DOG1* locus, some different primer sets were used to analyse promoter (P) and coding (C) regions of the *DOG1* gene by chromatin immunoprecipitation quantitative PCR (ChIP‐qPCR) in Arabidopsis seedlings (Fig. 1d). Significant HSI2 enrichment was detected at the proximal promoter regions (P1 and P2) of *DOG1* with the highest level of enrichment at P1. No significant enrichment of HSI2 was observed at the distal promoter (P3) or coding regions in the first and second exons (C1 and C2) of *DOG1*. To determine if transcription of *DOG1* is directly downregulated by HSI2, a luciferase reporter gene controlled by the *DOG1* promoter (*DOGpro:LUC*) was tested using transient expression assays and agroinfiltration of tobacco (*Nicotiana benthamiana)* leaves (Fig. 1e). Relatively strong luminescence signal, indicating substantial luciferase activity, and elevated levels of *LUC* mRNA were detected in leaves agroinfiltrated with *DOGpro:LUC* construct alone. However, luminescence and *LUC* mRNA expression from *DOGpro:LUC* was significantly reduced when co‐infiltrated with an HSI2‐expressing effector construct, indicating that expression from the *DOG1* promoter in tobacco leaves is repressed by co‐expression of HSI2.

### B3 domain is required for HSI2 function

**Fig. 2:**
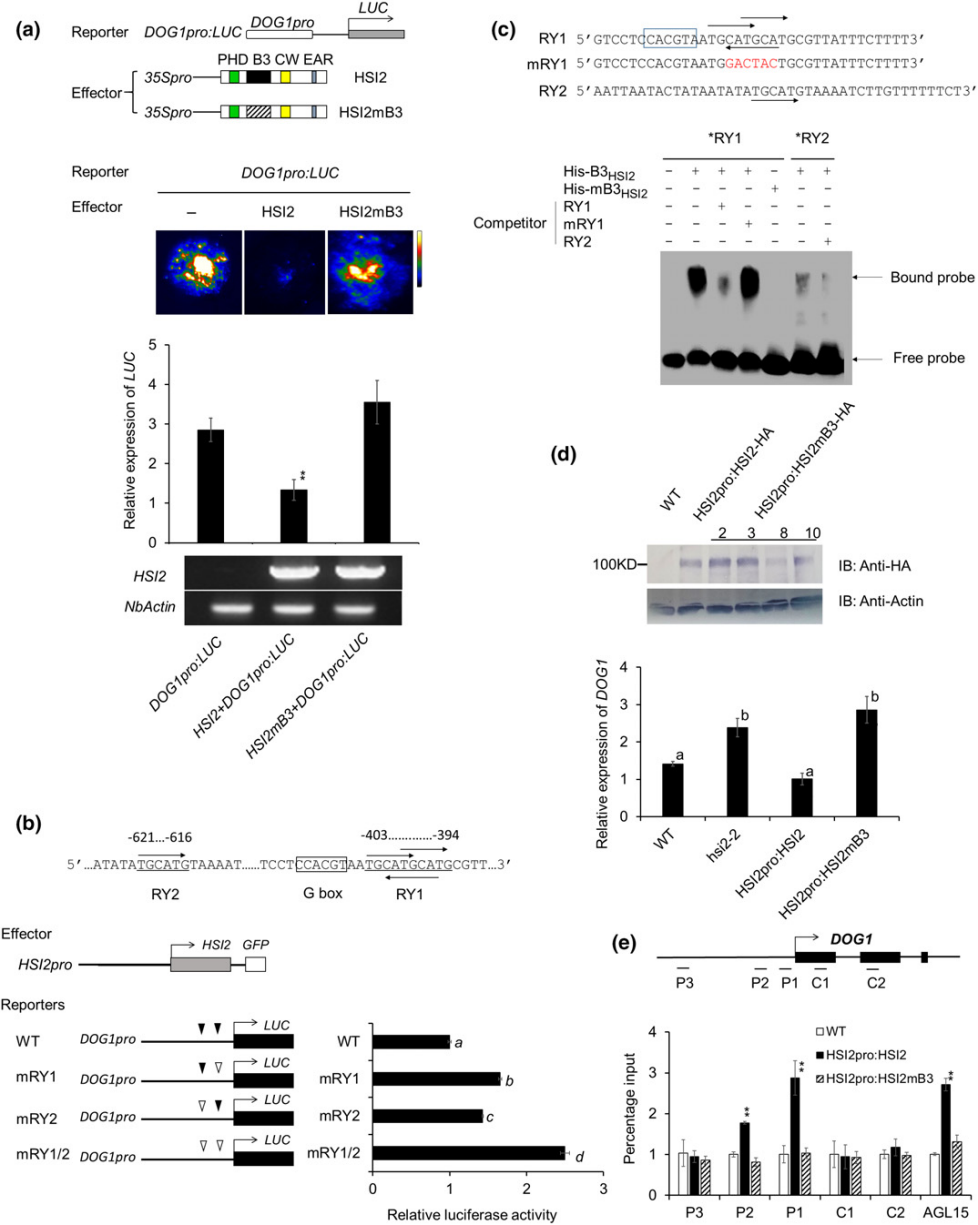
B3 domain is required for HSI2‐dependent repression of *DOG1.* (a) Schematic representation of reporter and effector constructs used to assay the function of the HSI2 B3 domain. In the reporter construct, a luciferase gene is driven by the *DOG1* promoter. In effector constructs, expression of the intact HSI2 or HSI2mB3 is controlled by the *CaMV 35S* promoter. Diagonal shading represents mutated B3 domain. Luminescence images and relative expression of the luciferase mRNA from *Nicotiana benthamiana* leaves co‐infiltrated with combinations of reporters and effectors, as indicated. *HSI2* and *NbActin* gene expression in infiltrated areas was assayed by RT‐qPCR. (b) Function of RY1 and RY2 elements in the repression of the *DOG1* promoter. Schematic representation of the locations of RY1, RY2 and G‐box sequence elements in the *DOG1* promotor are shown along with results from dual luciferase assays to evaluate the expression of reporter genes with intact or disrupted RY elements co‐expressed with HSI2 in Arabidopsis protoplasts. Solid triangles indicate RY element, open triangles indicate mutated RY element. Means of three independent experiments (±SD) are shown. Lowercase letters indicate significant differences (*P* < 0.01) between genotypes. (c) RY1, RY2 and mutant, mRY1 probes, shown with RY elements (arrows) and putative G‐box (boxed) indicated, used in EMSA assays with His‐B3_HSI2_ and mutant His‐mB3_HSI2_ polypeptides. Bands representing free and bound probe are indicated. (d) Expression of HA‐tagged HSI2mB3 protein was detected in transgenic Arabidopsis seedlings by immunoblot assays using anti‐HA antibody, anti‐actin was used to detect actin as a loading standard. Relative expression of *DOG1* in WT, *hsi2‐2*, *HSI2pro:HSI2‐HA* and *HSI2pro:HSI2mB3* Arabidopsis seedlings. RT‐qPCR assays were normalised by *EF1a*. Lowercase letters indicate significant differences (*P* < 0.01) between genotypes. (e) Enrichment of HSI2‐HA and HSI2mB3‐HA at the *DOG1* locus in transgenic Arabidopsis plants assayed by ChIP‐qPCR. Tested regions are indicated in the gene structure. Assay for enrichment at the *AGL15* locus was included as a positive control. Data represent means of three ChIP‐qPCR assays from three independent ChIP assays for each genotype. Error bars indicate SD. Asterisks indicate means significantly different from WT and *HSI2pro:HSI2mB3* at *P* < 0.01 (Student’s *t*‐test).

The B3 domain of HSI2 (B3_HSI2_) was reported to play an essential role in the regulation of *AGL15* and *FLC* by binding to RY elements in the proximal promoter or first intron of these genes, respectively (Qüesta *et al.*, 2016; Yuan *et al*., 2016; Chen *et al.*, 2018). We examined the function of the B3_HSI2_ domain in the regulation of the *DOG1* promoter by transient expression assays. An effector construct that encodes HSI2 with a mutated B3 domain (HSI2mB3) was co‐infiltrated with the *DOG1pro:LUC* reporter construct into *N. benthamiana* leaves (Fig. 2a). The *DOG1pro:LUC* reporter gene was strongly repressed by co‐expression with intact HSI2; however, co‐expression of HSI2mB3 resulted in high levels of reporter gene expression indicating that the B3 domain is required for HSI2‐dependent repression of the *DOG1* promoter.

Sequence analysis indicated that RY elements are distributed in the *DOG1* proximal promoter between −621 and −394 bp upstream of the transcriptional start site. The region corresponding to P1 in Fig. 2(b) contains overlapping RY elements (RY1, 5′‐TGCATGCATG‐3′), while the P2 region contains a single RY element (RY2, 5′‐TGCATG‐3′) (Fig. 2(b)). The expression of luciferase reporter genes under control of the intact *DOG1* promoter or promoters with disruptions in RY1, RY2 or both were tested in Arabidopsis protoplasts using a dual luciferase assay. As show in Fig. 2(b), loss of both RY1 and RY2 resulted in a 2.5‐fold increase in expression relative to the intact promoter, while mutation of RY1 led to a two‐fold increase and RY2 disruption gave a 1.5‐fold increase. Thus, the *DOG1* RY elements appear to act additively with the RY1 element showing a somewhat stronger repressive effect than RY2.

To test whether the B3_HSI2_ domain can bind directly to the RY elements in the *DOG1* promoter *in vitro*, we performed EMSA. RY1‐ and RY2‐containing DNA probes from the P1 and P2 regions of the *DOG1* promoter were labelled with biotin at the 3 end and incubated with His‐Trx‐tagged B3_HSI2_ fusion protein. A strong signal indicating binding of B3_HSI2_ to probes containing the complex RY1 sequence element was detected, but the signal for binding to probe containing the RY2 element was much weaker (Fig. 2c). Probe with a mutated RY1 element failed to bind B3_HSI2_, and a B3_HSI2_ polypeptide with a disrupted B3 domain (mB3_HSI2_) failed to bind the intact RY1 element‐containing probe. These results indicate that the B3 domain of HSI2 can specifically bind, *in vitro*, to the complex RY1 element in the proximal promoter of *DOG1*, while binding to the single RY element found in the P2 region was substantially lower, relative to RY1, under the conditions used.

To test whether the B3 domain is required for HSI2 enrichment at the *DOG1* locus, we carried out ChIP‐qPCR assays using a transgenic Arabidopsis line that expressed HSI2 with a disrupted B3 domain under the control of the native *HSI2* promoter in the *hsi2‐2* mutant background (*HSI2pro:HSI2mB3*) (Chen *et al.*, 2018). Plants from three independent transgenic lines (2, 3 and 10) that express HSI2mB3 at levels similar to the intact *HSI2* transgene were analysed as biological replicates (Fig. 2d). Expression of *DOG1* remained high in these lines, indicating that HSI2‐dependent repression of *DOG1* was not restored by complementation with HSI2mB3. ChIP‐qPCR analysis showed that, unlike intact HSI2, HSI2mB3 fails to accumulate at the proximal promoter region of the *DOG1* locus (Fig. 2e). Similar results were obtained for the enrichment of HSI2 at the *AGL15* locus, which was included as a positive control in this ChIP‐qPCR analysis.

### PHD domain is required for HSI2‐mediated repression of *DOG1*

**Fig. 3:**
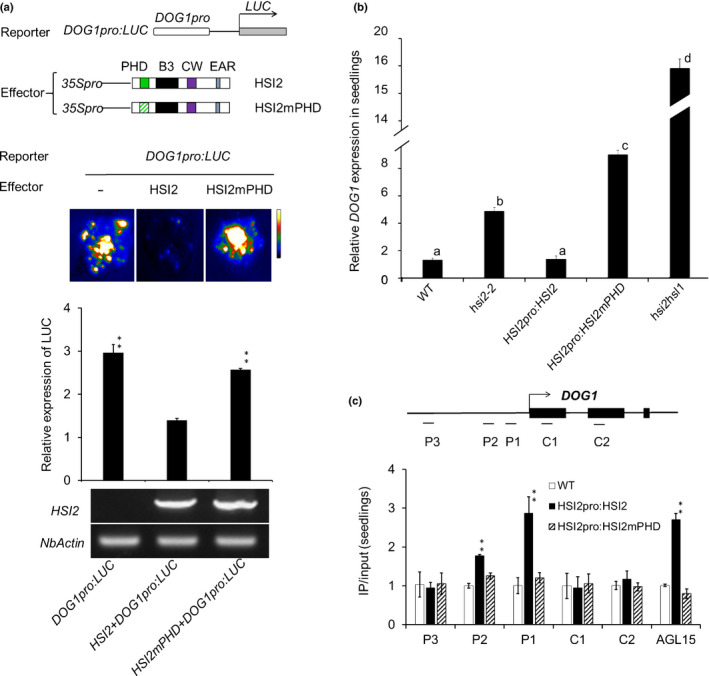
HSI2 PHD domain is essential for the repression of *DOG1*. (a) Schematic representation of reporter and effector constructs used to test the function of the HSI2 PHD domain. In effector constructs, green diagonal shading represents a mutated PHD domain. Luminescence image and relative expression of luciferase reporter genes from *Nicotiana benthamiana* leaves co‐infiltrated with combinations of reporter and effector constructs, as indicated. *HSI2* and *NbActin* gene expression in each infiltrated area was assayed by RT‐qPCR. (b) Relative expression of *DOG1* in Arabidopsis seedlings of wild‐type (WT), *hsi2‐2*, *HSI2pro:HSI2‐HA* and *HSI2pro:HSI2mPHD.* RT‐qPCR assays were normalised using *EF1a*. Lowercase letters indicate significant differences (*P* < 0.01) between genetic backgrounds. (c) Results of ChIP‐qPCR analyses to compare the enrichment of HSI2‐HA and HSI2mPHD‐HA at the *DOG1* locus in Arabidopsis plants. Tested regions are indicated in the gene structure. *AGL15* assay was included as a positive control. Data represent the means of three ChIP‐qPCR assays from three biological replicates. Error bars indicate SD. Asterisks indicate means significantly different from control plants (WT) at *P* < 0.01.

The HSI2 PHD domain (PHD_HSI2_) was shown to be essential for HSI2‐dependent repression of *AGL15* expression and HSI2 enrichment at the *AGL15* locus (Chen *et al.*, 2018). To test whether PHD_HSI2_ also plays a role in *DOG1* repression, the *DOG1pro:LUC* reporter construct was co‐infiltrated into *N. benthamiana* leaves with intact HSI2 or HSI2mPHD in which eight conserved amino acids of the PHD domain were substituted (C39S, C42S, C65S, C68S, H73L, C76S, C93S and C96S) (Chen *et al.*, 2018). As expected, luciferase activity and mRNA expression levels were significantly reduced when *DOG1pro:LUC* was co‐expressed with intact HSI2, but co‐expression with HSI2mPHD had no effect on reporter gene expression (Fig. 3a). Transgenic complementation of *hsi2‐2* plants with HSI2mPHD also failed to repress *DOG1* expression (Fig. 3b). Rather, expression of HSI2mPHD in *hsi2‐2* plants resulted in an increase in *DOG1* expression by more than two‐fold, relative to the *hsi2‐2* null allele, although expression in these plants was not as high as in *hsi2 hsl1* double mutant seedlings (Fig. 3b). To test if the PHD domain is also required for HSI2 enrichment at the *DOG1* locus, we performed ChIP‐qPCR using WT, and *HSI2pro:HSI2*‐ and *HSI2pro:HSI2mPHD*‐expressing Arabidopsis seedlings (Fig. 3c). The results showed that, relative to intact HSI2, HSI2mPHD failed to accumulate at the P1 or P2 regions of the *DOG1* gene, indicating that the PHD domain is essential for HSI2 accumulation at the *DOG1* locus.

### CW and EAR domains are not required for HSI2‐dependent repression of *DOG1*

Chen *et al. *(2018) reported that HSI2‐mediated repression of *AGL15* expression requires the EAR motif but not the CW domain. To investigate whether CW and EAR domains of HSI2 contribute to the repression of *DOG1*, we tested the functions of HSI2 with disrupted CW or deleted EAR domains in the regulation of the *DOG1* promoter by transient expression in *N. benthamiana* leaves (Fig. S1a). Similar to intact HSI2, co‐expression of HSI2mCW or HSI2‐ΔEAR effector constructs strongly repressed luciferase activity and decreased mRNA levels from the *DOG1pro:LUC* reporter construct, indicating that the CW and EAR domains are not required for HSI2‐dependent repression of the *DOG1* promoter in agroinfiltrated leaves (Fig. S1b). Relative expression of endogenous *DOG1* was also tested in *HSI2pro:HSI2mCW* and *HSI2pro:HSI2mEAR* rescued *hsi2‐2* Arabidopsis seedlings^27^. Consistent with the agroinfiltration assays, the results showed that *DOG1* expression in these mutant lines was similar to that in WT seedlings, being strongly repressed relative to *hsi2‐2* (Fig. S1c), confirming that intact HSI2 CW and EAR domains are not required for HSI2‐mediated repression of *DOG1* expression in Arabidopsis.

### HSI2 and HSL1 form dimers *in vivo*

**Fig. 4:**
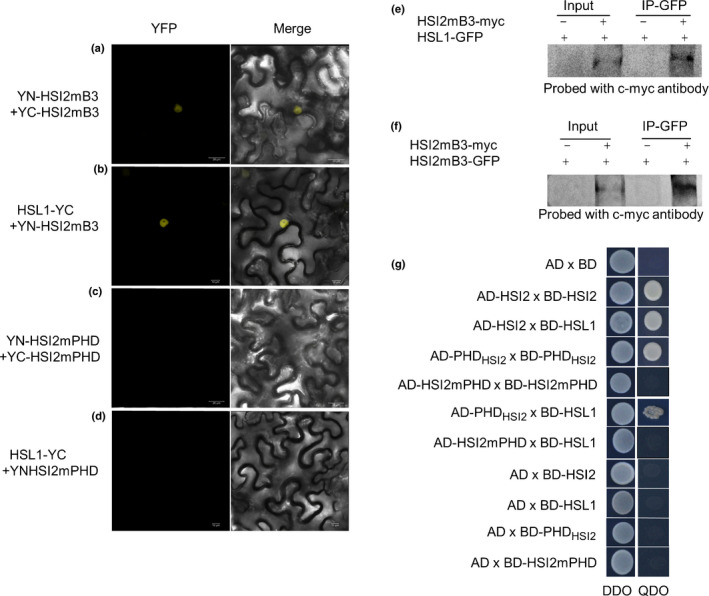
Dimerisation between HSI2 and HSL1 is dependent on PHD‐like domain. Bimolecular fluorescence complementation (BiFC) analysis of protein–protein interactions between (a) HSI2mB3:HSI2mB3, (b) HSI2mB3:HSL1, (c) HSI2mPHD:HSI2mPHD and (d) HSI2mPHD:HSL1 using *Nicotiana benthamiana* leaves co‐infiltrated with combinations of corresponding constructs. (e) Co‐immunoprecipitation (Co‐IP) showing interaction between HSI2mB3 and HSI2mB3 and (f) HSI2mB3 and HSL1. Total protein was extracted from agroinfiltrated tobacco leaves co‐expressing *35S:HSI2mB3‐GFP* and *35S:HSI2mB3‐myc*, *35S:HSL1‐GFP* and *35S: HSI2mB3‐myc*, only *35S:HSI2mB3‐GFP* or only *35S:HSL1‐GFP*. HSI2mB3‐GFP and HSL1‐GFP was immunoprecipitated with anti‐GFP antibody and immunoblots were probed with anti‐c‐myc antibody. (g) Yeast‐two‐hybrid interaction assays. Yeast cells transformed with indicated genes were selected on Double Dropout medium (DDO, lacking Leu and Trp) and quadruple dropout medium (QDO, lacking adenine, His, Leu and Trp) media.

Chhun *et al. *(2016) reported the formation of HSI2:HSI2 and HSL1:HSL1 homodimers, along with HSI2:HSL1 heterodimers. To confirm these findings, we tested the interactions between HSI2 and HSL1 using both BiFC and yeast‐two‐hybrid assays, which showed both heterodimer and homodimer formation (Figs 4g, S2a–c). Further analysis indicated that the B3 domain is not required for dimerisation, as HSI2mB3 was still able to form homodimers (HSI2mB3:HSI2mB3), and heterodimers (HSI2mB3:HSL1) *in vivo* (Fig. 4a,b). However, HSI2mPHD was unable to interact with either HSI2 or HSL1 (Fig. 4c,d,g). These BiFC results were confirmed using both co‐immunoprecipitation (Co‐IP) (Fig. 4e,f) and yeast‐two‐hybrid assays (Fig. 4g).

### HSL1 enrichment at the *DOG1* promoter is independent of HSI2

**Fig. 5:**
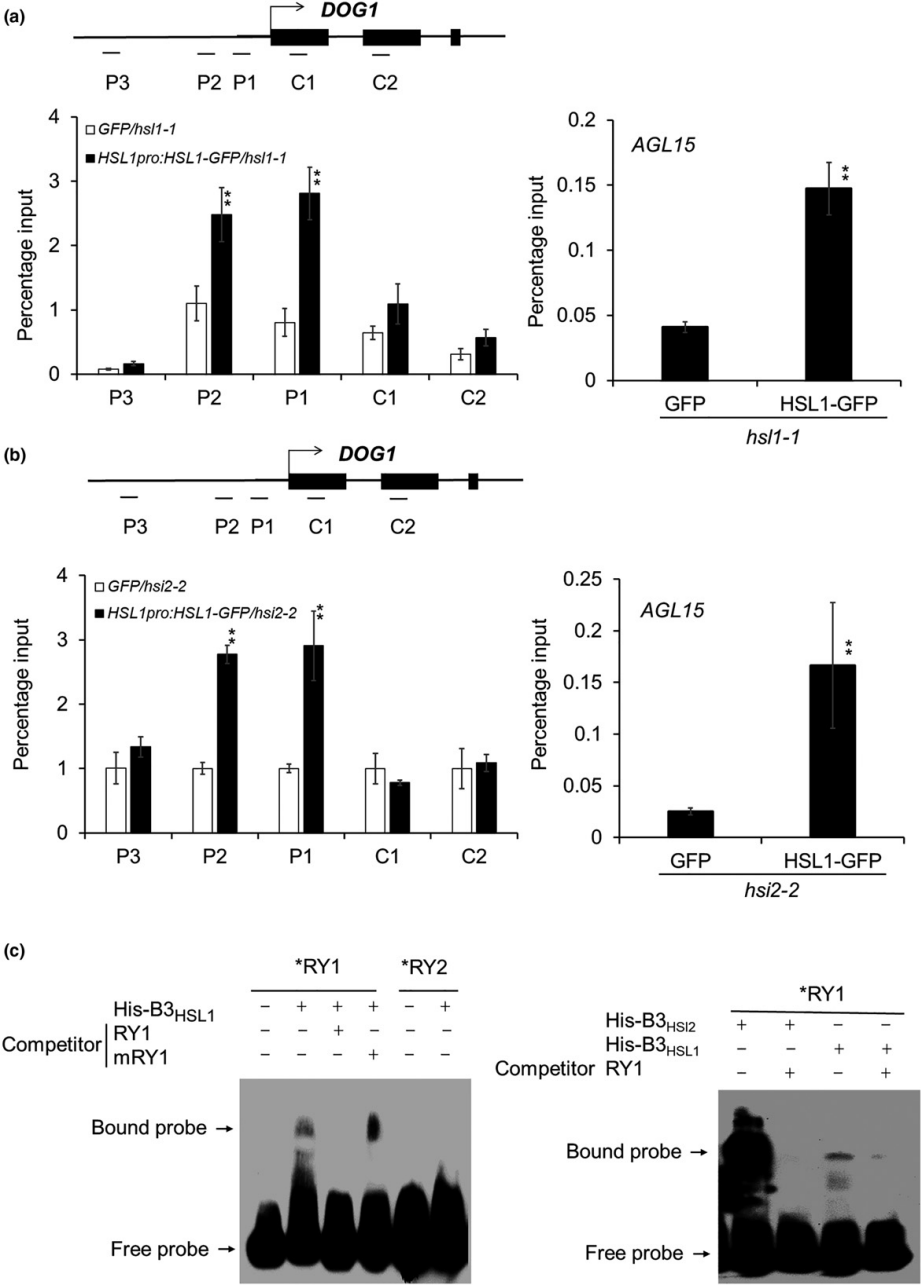
HSL1 directly binds to the *DOG1* promoter. (a, b) ChIP‐qPCR analysis of HSL1 enrichment at *DOG1* locus in *hsl1‐1* and *hsi2‐2* in Arabidopsis protoplasts. *HSL1‐GFP* was expressed under the control of the native *HSL1* promoter. Tested regions are indicated in the gene structure. ChIP‐qPCR assays showing enrichment of HSL1 at the *AGL15* locus in both *hsl1‐1* and *hsi2‐2* genetic backgrounds are included as positive controls. Data represent the means of three assays from three biological replicates. Error bars indicate SD. Asterisks indicate means significantly different from control at *P* < 0.01. (c) EMSA assay for the detection of DNA–protein interaction between RY1 and RY2, and mutant mRY1 probes with His‐B3_HSL1_ polypeptide. Bands representing free and bound probe are indicated. The stronger EMSA signal seen when using the B3_HSI2_ polypeptide than with the B3_HSL1_ polypeptide indicates that the B3_HSI2_ domain may have higher affinity for the RY1 probe than the B3_HSL1_ domain.

Protein sequence analysis showed that HSI2 and HSL1 share high sequence identity and contain similar conserved regions (Tsukagoshi *et al.*, 2007). As HSI2 harbours both PHD and B3 domains, which are required for HSI2 accumulation at the *DOG1* promoter (Figs 2e, 3c) and are essential for full repression of *DOG1* expression, we predicted that HSL1 might also be enriched in the chromatin of the *DOG1* locus. To confirm this, a gene construct that expresses GFP‐tagged HSL1 under the control of the native *HSL1* promoter (*HSL1pro:HSL1‐GFP*) was transformed into the *hsl1‐1* Arabidopsis protoplast for ChIP‐qPCR assays. The results indicated that, like HSI2, HSL1 is significantly enriched at the proximal promoter regions (P1 and P2) of the *DOG1* locus (Fig. 5a). To test whether the enrichment of HSL1 at the *DOG1* locus depends on HSI2, ChIP‐qPCR was also performed in protoplasts from *hsi2‐2* Arabidopsis plants that transiently expressed HSL1‐GFP. In these assays, HSL1 was still able to accumulate at the proximal promoter region of *DOG1* in the absence of HSI2, indicating that HSL1 enrichment at *DOG1* is independent of HSI2 (Fig. 5b). Significant HSI2‐independent enrichment of HSL1 was also detected at the *AGL15* promoter (Fig. 5a,b). To confirm the hypothesis that HSL1 can physically bind to RY elements in the *DOG1* promoter *in vitro*, we performed EMSA assays using His‐tagged B3_HSL1_ fusion protein incubated with biotin‐labelled probes containing RY1 and RY2 from the P1 and P2 regions of the *DOG1* promoter, respectively (Fig. 5c). Consistent with the binding activity of the B3_HSI2_ polypeptide, the B3_HSL1_ polypeptide can bind to a probe that contains the complex RY1 element more effectively than to a probe containing the single RY2 element (Fig. 5c). However, EMSA signals for B3_HSL1_ binding to RY1 in these assays was much weaker than for B3_HSI2_. These results demonstrate that the B3 domain of HSL1 can bind to RY elements in the proximal promoter region of *DOG1*, leading to HSI2‐independent HSL1 enrichment at the *DOG1* locus. However, the reduced EMSA signal for binding of B3_HSL1_ appears to indicate that its affinity for RY elements is reduced relative to the B3_HSI2_.

### Repression of *DOG1* by HSI2 and HSL1 is associated with H3K27me3 enrichment

**Fig. 6:**
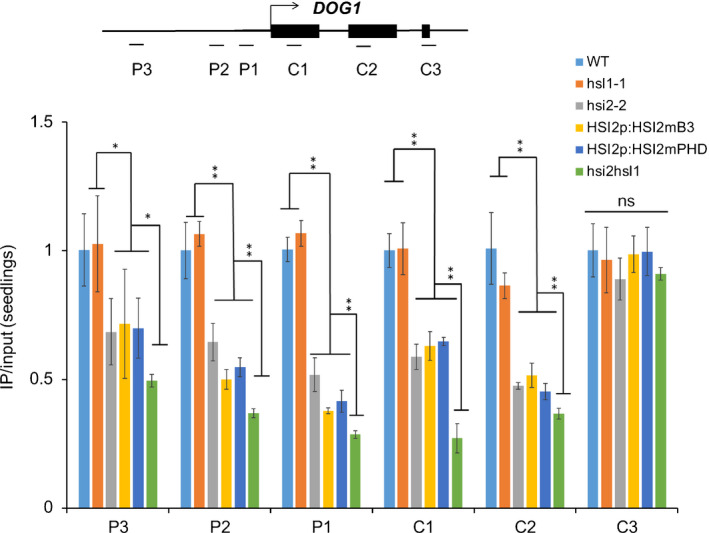
Both HSI2 and HSL1 are involved in deposition of H3K27me3 marks at *DOG1* locus. ChIP‐qPCR analysis of H3K27me3 enrichment at the *DOG1* locus in wild‐type (WT), *hsl1‐1*, *hsi2‐2*, *HSI2pro:HSI2mPHD*, *HSI2pro:HSI2mB3*, *hsi2 hsl1* Arabidopsis seedlings. Tested regions are indicated in the gene structure. Data represent means of three assays from three biological replicates. Error bars indicate SD. *, *P* < 0.05; **, *P* < 0.01; ns, nonsignificant.

Previously, we showed that loss of HSI2 in *hsi2‐2* knockout and disruption in *hsi2‐4* PHD domain Arabidopsis mutant seedlings resulted in de‐repression of *DOG1* expression and reduced deposition of H3K27me3 at the *DOG1* locus (Veerappan *et al.*, 2012, 2014). As HSL1 also targets *DOG1*, we used ChIP‐qPCR to examine the effects of various *hsi2* and *hsl1* Arabidopsis mutations on the levels of H3K27me3 at *DOG1* (Fig. 6). While enrichment of H3K27me3 was significantly reduced, relative to WT, across the *DOG1* locus in three *hsi2* mutant lines (*hsi2‐2*, *HSI2mPHD* and *HSI2mB3*), H3K27me3 levels in *hsl1‐1* seedlings were similar to WT. These results indicated that intact HSI2 mediates the trimethylation of H3K27 at WT levels in the absence of HSL1 and the PHD and B3 domains are required for this activity. Thus, as with transcriptional repression, HSI2 is able to fully complement the loss of HSL1 in H3K27me3 deposition. However, H3K27me3 deposition at the *DOG1* locus was more strongly reduced in the *hsi2 hsl1* double mutant, than in the three *hsi2* mutants, with the greatest effect seen at the C1 region (Fig. 6). Thus, it is apparent that HSL1 contributes to the deposition of H3K27me3 at this locus.

### LHP1 and CLF interact with both HSI2 and HSL1 to regulate *DOG1* expression through deposition of H3K27me3

**Fig. 7:**
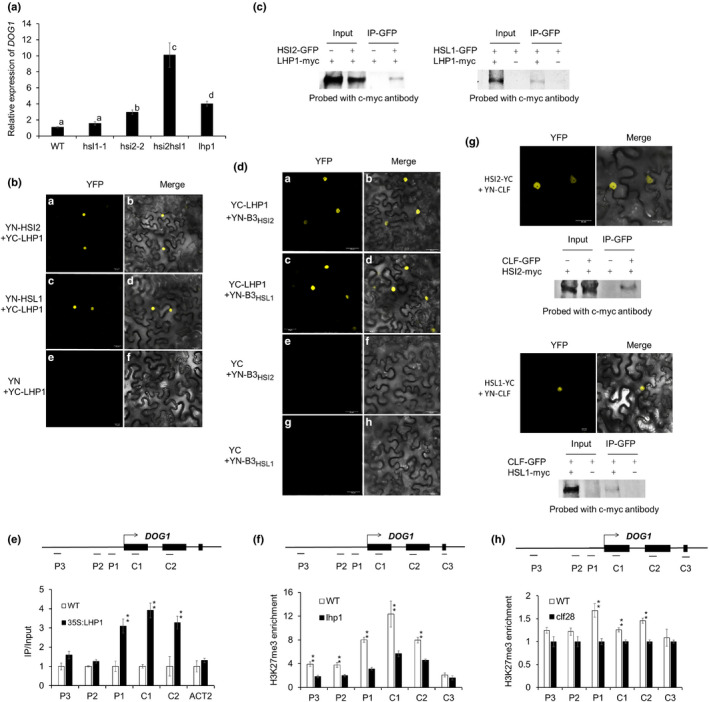
LHP1 and CLF interact with both HSI2 and HSL1. (a) Relative expression of *DOG1* in freshly harvested seeds from wild‐type (WT), *hsl1‐1*, *hsi2‐2*, *hsi2 hsl1* Arabidopsis plants. RT‐qPCR assays were normalised by *EF1a*. Lowercase letters indicate significant differences *P* < 0.01. (b) BiFC analysis using *Nicotiana benthamiana* leaves showing LHP1:HSI2 and LHP1:HSL1 interactions. The N‐terminal region of enhanced yellow fluorescent protein (EYFP) was fused to HSI2 (YN‐HSI2) or HSL1 (YN‐HSL1), and C‐terminal region of EYFP was fused to LHP1 (YC‐LHP1). EYFP fluorescence (YFP) and EYFP fluorescence images merged with bright‐field images (Merge) are shown. Empty plasmid containing YN‐YFP was used as a negative control (e, f). (c) Co‐IP showing interaction of LHP1/HSI2, and LHP1/HSL1. Total protein was extracted from tobacco leaves co‐expressing *35S:HSI2‐GFP* and *35S:LHP1‐myc*, *35S:HSL1‐GFP* and *35S:LHP1*, *35S:HSL1‐GFP* alone or *35S:LHP1‐myc* alone. HSI2‐GFP and HSL1‐GFP was immunoprecipitated with anti‐GFP antibody, and the immunoblot was probed with anti‐c‐*myc* antibody. (d) BiFC analysis using *N. benthamiana* leaves showing that LHP1 interacts with the B3 domains of HSI2 (B3_HSI2_) and HSL1 (B3_HSL1_). (e) Results of ChIP‐qPCR analysis of LHP1 enrichment at *DOG1* locus in WT and *35S:LHP1‐myc* Arabidopsis seedlings. Tested regions are indicated in the gene structure. *ACT2* was included as a control. Data represent means of three assays from three biological replicates. Error bars indicate SD. Asterisks indicate means significantly different from control (WT) *P* < 0.01. (f) Results of ChIP‐qPCR analysis of H3K27me3 enrichment at *DOG1* locus in WT and *lhp1* Arabidopsis seedlings. (g) BiFC and Co‐IP from *N. benthamiana* leaves showing interaction of CLF/HSI2, and CLF/HSL. (h) Results of ChIP‐qPCR analysis of H3K27me3 enrichment at *DOG1* locus in WT and *clf28* Arabidopsis seedlings.

Yuan *et al*. (2016) reported that LHP1, which may serve as a bridge between PRC2 and PRC1 (Xu & Shen, 2008; Bratzel *et al.*, 2010), is recruited by HSI2 to the *FLC* locus to promote deposition of H3K27me3, leading to downregulation of *FLC* expression. Therefore, it seemed likely that LHP1 could also be involved in the downregulation of *DOG1* expression. To test this possibility, we evaluated the role of LHP1 in the repression of *DOG1*. Our results show that *DOG1* expression is significantly upregulated in *lhp1* Arabidopsis seeds, relative to that in WT, *hsl1‐1* and *hsi2‐2*, but remained lower than in the *hsi2 hsl1* double mutant (Fig. 7a). Protein–protein interactions between HSI2:LHP1 and HSL1:LHP1 *in vivo* were confirmed by BiFC and Co‐IP assays (Fig. 7b,c) and BiFC analysis also showed that the B3_HSI2_ and B3_HSL1_ domains are sufficient for interaction with LHP1 (Fig. 7d). ChIP‐qPCR analysis using transgenic Arabidopsis seedlings that express Myc‐LHP1 showed significant LHP1 enrichment at both the proximal promoter (P1) and exons (C1 and C2) of the *DOG1* locus (Fig. 7e). Finally, ChIP‐qPCR analysis in *lhp1* mutant seedlings showed significant reductions in H3K27me3 chromatin marks across the *DOG1* locus, relative to WT (Fig. 7f). Shu *et al. *(2019) reported that CLF, a histone methyltransferase component of PRC2, accumulated at *FLC*, *AGL15* and *DOG1* genes that are directly regulated by HSI2 (Qüesta *et al.*, 2016; Yuan *et al*., 2016; Chen *et al.*, 2018). We used BiFC and Co‐IP assays to confirm that CLF interacts with HSI2 and HSL1 *in vivo* (Fig. 7g) and ChIP‐qPCR analysis of H3K27me3 deposition in *clf28* mutant seedlings showed significant decreases of this histone mark at P1, C1 and C2 regions of the *DOG1* locus compared with WT (Fig. 7h). The relatively small effect on H3K27me3 in *clf28* could be due to the redundancy of CLF with the other PRC2 histone methyltransferase subunits SWINGER and MEDEA.

## Discussion

We identified *DOG1,* a key regulator of seed dormancy in Arabidopsis (Alonso‐Blanco *et al.*, 2003; Bentsink *et al.*, 2006, 2010; Huang *et al.*, 2010), as a direct regulatory target of HSI2/HSL1‐mediated transcriptional repression. HSI2 has been shown to regulate the developmental transition from seeds to seedlings, at least in part, by directly silencing the expression of the embryogenesis‐promoting gene *AGL15* (Chen *et al.*, 2018). HSI2 also affects the switch from vegetative growth to flowering by the downregulation of *FLC* and *FLOWERING LOCUS T* (*FT*) (Qüesta *et al.*, 2016; Yuan *et al*., 2016; Jing *et al.*, 2019). In these cases, HSI2 was shown to interact, through its B3 DNA binding domain, with a pair of RY elements, located in the proximal promoters of *AGL15*, the first intron of *FLC* or the second intron of *FT*. We show here that HSI2 is also enriched in the chromatin at the proximal 5 region of the *DOG1* locus in Arabidopsis seedlings and binds strongly, *in vitro*, to the complex RY1 region in the proximal *DOG1* promoter, which contains multiple overlapping canonical RY elements, and more weakly to the RY2 region, which contains a single RY element. (Fig. 2b). Transient reporter gene expression analysis indicates that both RY1, and the simpler RY2 element contribute to transcriptional repression and these observations agree with our ChIP‐qPCR data that show strong enrichment of HSI2 and HSL1 *in vivo* at the P1 region, which contains the complex RY element, with lower but still significant enrichment at the P2 region, which contain the simple RY2 sequence. As with *AGL15* (Chen *et al.*, 2018), the conserved HSI2 B3 domain is required for HSI2‐dependent silencing of the *DOG1* promoter and enrichment of HSI2 at the *DOG1* locus *in vivo*.

Analysis of native *DOG1* expression in seeds and seedlings of *hsi2* and *hsl1* Arabidopsis knock out mutant lines indicates that *HSI2* and *HSL1* act redundantly in the regulation of *DOG1* expression (Fig. 1a). While loss of HSL1 does not significantly affect *DOG1* expression, loss of HSI2 results in a three‐fold increase in expression of this gene. However, loss of both HSI2 and HSL1 leads to *c.* 14‐fold increase in *DOG1* expression and a significant increase in seed dormancy was seen in the *hsi2 hsl1* double mutant. These results indicate that both HSI2 and HSL1 are necessary for full transcriptional repression of *DOG1* and suggest that the relatively high levels of *DOG1* expression in the double mutant lead to increased seed dormancy.

Our previously published data indicated that the PHD‐like domain also plays a critical functional role in HSI2‐mediated transcriptional silencing (Veerappan *et al.*, 2012, 2014; Chen *et al.*, 2018). As shown in Fig. 2(d), expression of HSI2mB3 in the *hsi2‐2* seedlings fails to rescue the transcriptional repression of *DOG1*, resulting in expression levels similar to that in *hsi2‐2* plants. Conversely, expression of the HSI2mPHD mutant in the *hsi2‐2* knock out mutant background results in *DOG1* expression reaching levels *c.* two‐fold higher than in *hsi2‐2* seedlings, although not as high as in the *hsi2 hsl1* double mutant (Fig. 3b). This dominant‐negative effect indicates that the PHD‐like domain plays an important role in the functional interaction between HSI2 and HSL1. We confirmed that HSI2 and HSL1 can form homodimers and heterodimers *in vivo*, and the HSI2 PHD‐like domain is required for dimerisation. Therefore, it seems likely that the inability of HSI2mPHD subunits to dimerise interferes with HSL1 activity. One possible explanation for this could be competition between functionally intact HSL1 dimers and HSI2mPHD monomers, which have intact B3 domains and may retain the ability to bind nonproductively to RY elements in the *DOG1* promoter. However, this seems unlikely since HSI2mPHD is not enriched at the *DOG1* locus (Fig. 3c). Alternatively, HSI2mPHD monomers may compete with HSL1 dimers for components of the HSI2/HSL1 repressive complex such as PRC2 subunits, LHP1 and histone deacetylases.

*DOG1* expression is controlled by complex and diverse mechanisms involving alternative splicing, alternative polyadenylation, histone modifications, and a *cis*‐acting antisense noncoding transcript known as *asDOG1* (Bentsink *et al.*, 2006; Müller *et al.*, 2012; Graeber *et al.*, 2014; Cyrek *et al.*, 2016; Fedak *et al.*, 2016). Developmental regulation of *DOG1* expression during seed development depends on the LAFL network of master transcription factors that regulate seed maturation. These include a homologue of the NUCLEAR TRANSCRIPTION FACTOR Y subunit B known as LEAFY COTYLEDON1 (LEC1), along with ABSCISIC ACID INSENSITIVE3 (ABI3), FUSCA3 (FUS3), and LEC2, which, like HSI2, contain conserved, plant‐specific B3 DNA binding domains that interact with RY *cis*‐acting elements (Giraudat *et al.*, 1992; Meinke, 1992; Keith *et al.*, 1994; West *et al.*, 1994; Lotan *et al.*, 1998; Luerssen *et al.*, 1998; Stone *et al.*, 2001). Bryant *et al. *(2019) recently reported that increased expression of *DOG1* under low‐temperature conditions is directly regulated by bZIP67, and indirectly by LEC1. These authors showed that bZIP67 binds to G‐box‐like elements in the *DOG1* promoter, one of which is located just upstream of RY1 (Fig. 2b) (Bryant *et al.*, 2019). Disruption of this complex RY1 element in the *DOG1* promoter was reported to completely abolish its ability to drive *GUS* expression in protoplasts, suggesting that it is critical both for upregulation and downregulation of *DOG1* expression (Bryant *et al.*, 2019). It is possible that the RY1 element could be bound by one or more of the LEC1‐induced B3 domain‐containing AFL factors ABI3, FUS3 or LEC2 (Stone *et al.*, 2001; Braybrook *et al.*, 2006; Baud *et al.*, 2016) and is required for developmental regulation of *DOG1* expression (Braybrook *et al.*, 2006; Mönke *et al.*, 2012; Wang & Perry, 2013; Baud *et al.*, 2016; González‐Morales *et al.*, 2016; Pelletier *et al.*, 2017). This conclusion is supported by the results of ChIP experiments that showed that FUS3 binds to the *DOG1* locus *in vivo* (Wang & Perry, 2013). However, results from our transient expression assays using luciferase reporter genes controlled by intact and RY element‐disrupted *DOG1* promoters showed that, rather than abolish promoter activity, loss of one or both RY elements resulted in increased reporter gene expression in protoplasts (Fig. 3b). Therefore, the potential regulatory relationship between HSI2/HSL1 and AFL factors at the RY elements in the *DOG1* promoter remains to be elucidated.

Changes in chromatin structure associated with histone modifications play a critical role in the regulation of seed dormancy (Footitt *et al.*, 2015). Repressive H3K27me3 marks form along the *DOG1* gene as dormancy declines and these marks accumulate rapidly on exposure to light. Our data support the hypothesis that HSI2 and HSL1 contribute to the repression of *DOG1* expression primarily through PRC2‐dependent deposition of H3K27me3 at the *DOG1* locus. Compared with WT, three *hsi2* mutant lines, including the T‐DNA knockout *hsi2‐2*, and *hsi2‐2* complemented with either *HSI2pro:HSI2mB3* or *HSI2pro:HSI2mPHD*, showed similar decreases in H3K27me3 levels at the *DOG1* locus. Although H3K27me3 at the *DOG1* locus in *hsl1* mutant seedlings was not significantly reduced relative to WT, H3K27me3 in *hsi2 hsl1* double mutant seedlings was substantially lower than in *hsi2* mutants. Therefore, although HSI2 is able to fully complement the *DOG1* expression and H3K27 trimethylation phenotypes in *hsl1* knockout seedlings, both HSI2 and HSL1 contribute to the deposition of H3K27me3 and transcriptional repression at the *DOG1* locus. Complementation of *hsi2‐2* with *HSI2pro:HSI2mPHD* had a dominant‐negative effect on the repression of *DOG1* expression (Fig. 3b) but trimethylation of H3K27 in this line was not significantly different from *hsi2‐2*. Therefore, it is possible that the HSI2 PHD domain is required to mediate, directly or indirectly, transcriptional repression mechanisms other than the deposition of H3K27me3. For example, the histone deacetylases HDA6 and HDA19 were reported to interact with HSI2 and HSL1, respectively (Zhou *et al.*, 2013; Chhun *et al.*, 2016), and Zeng *et al. *(2019) recently reported that HDA9 is required for polycomb‐dependent silencing of *FLC*. These authors proposed that deacetylation of H3K27 by HDA9 may be necessary for its subsequent methylation by PRC2. Results reported by van Zanten *et al. *(2014) suggest that HDA9 negatively affects germination and is involved in the suppression of seedling traits in seeds, which is the opposite of that reported for HDA6 and HDA19, which repress embryonic characteristics in seedlings.

HSI2 directly interacts with the core PRC2 subunit MSI1 (Chen *et al.*, 2018) and, as shown in Fig. 7, both HSI2 and HSL1 also interact with CLF and LHP1. LHP1 interacts with PRC2 components, including MSI1, and is able to recognise H3K27me3 and spread this repressive histone mark throughout the Arabidopsis genome (Turck *et al*, 2007; Zhang *et al*, 2007; Exner *et al.*, 2009; Derkacheva *et al.*, 2013). HSI2 recruits LHP1 to the *FLC* locus (Yuan *et al*., 2016) and our results show that *DOG1* expression is de‐repressed in *lhp1* seedlings (Fig. 7a). LHP1 is enriched at the *DOG1* locus (Fig. 7e), and is required for deposition of H3K27me3 chromatin marks (Fig. 7f). Thus, it is clear that LHP1 plays an important role in the repression of *DOG1* and it is possible that LHP1 may provide a bridge between H3K27me3 marks and the HSI2/HSL1/PRC2 complex.

**Fig. 8:**
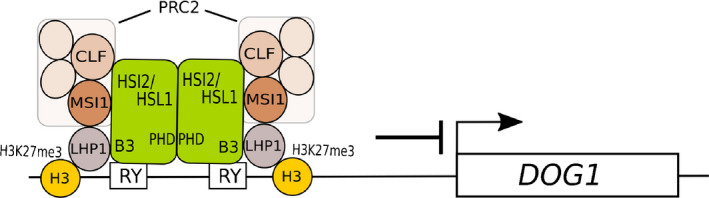
The proposed model for the repression of *DOG1* expression by HSI2 and HSL1. Simplified model showing the assembly of a hypothetical HSI2/HSL1 repressive complex at the *DOG1* locus. PHD domain‐dependent HSI2/HSL1 dimerisation allows for B3 domain‐dependent binding to RY elements located upstream of the *DOG1* transcription start site (arrow). Direct interaction with LHP1 could provide links to H3K27me3 marks associated with nearby nucleosomes and PRC2 probably recruited through direct contacts with MSI1 and CLF subunits. The resulting complex, possibly along with other components, catalyses the accumulation of additional H3K27me3 marks that repress *DOG1* expression, leading to the release of seed dormancy.

Based on our ChIP and protein interaction data, we propose a hypothetical model for the HSI2‐ and HSL1‐mediated downregulation of *DOG1* expression in seedlings, as shown in Fig. 8. Repression of *DOG1* is mediated directly by the combined action of the closely related transcriptional repressors HSI2 and HSL1, which form dimers that bind, via their conserved B3 domains, to RY *cis*‐acting elements located upstream of the transcription start site. Conserved PHD‐like domains are necessary for dimer formation and are required for transcriptional silencing activity. Direct contacts with LHP1 and PRC2 subunits MSI1 and CLF lead to the formation of a repressive complex that mediate the accumulation and spread of H3K27me3 chromatin marks at the *DOG1* locus, leading to decreased *DOG1* expression. These data do not discount to potential functions of other transcriptional repressive cofactors, such as histone deacetylases or MED13, in HSI2/HSL1‐dependant silencing of *DOG1* and other direct target genes. The reduced germination of *hsi2 hsl1* double mutant seeds suggests that at least one of these factors contributes to the release of seed dormancy and, as *DOG1* expression is also repressed by HSI2/HSL1 in seedlings, it seems likely that they are involved in maintaining low levels of *DOG1* expression during postgerminative growth and development, possibly affecting drought tolerance and flowering (Huo *et al*, 2016; Yatusevich *et al.*, 2017).

## Author contributions

The research was performed and analysed primarily by NC, and he and RDA conceived the research, designed experiments and wrote the manuscript. Specific experiments were performed by HW and HA. VV and MT provided critical analysis of the research and editing of the manuscript.

## Supporting information


**Dataset S1 **Table listing all of the primers used for RT‐qPCR, ChIP‐qPCR, DNA fragment cloning, site directed mutagenesis and EMSA in this study.

**Fig. S1 **Disruption of CW and EAR domains does not affect HSI2‐mediated regulation of *DOG1* expression.
**Fig. S2 **HSI2 and HSL1 form homodimers and heterodimers *in vivo*.
Please note: Wiley Blackwell are not responsible for the content or functionality of any Supporting Information supplied by the authors. Any queries (other than missing material) should be directed to the *New Phytologist* Central Office.
