# Supplementary material for: HSI2/VAL1 and HSL1/VAL2 function redundantly to repress DOG1 expression in Arabidopsis seeds and seedlings
Source: New Phytol. 2020 Apr 25;227(3):840–56. doi: 10.1111/nph.16559 (PMC7383879; doi:10.1111/nph.16559)
Supplement: Supplementary file 1 — Dataset S1 Table listing all of the primers used for RT‐qPCR, ChIP‐qPCR, DNA fragment cloning, site directed mutagenesis and EMSA in this study. [file NPH-227-840-s001.pdf]

New Phytologist Supporting Information

HSI2/VAL1 and HSL1/VAL2 function redundantly to repress DOG1 expression in Arabidopsis seeds and seedlings

Naichong Chen, Hui Wang, Haggag Abdelmageed, Vijaykumar Veerappan, Million Tadege, and Randy D. Allen

Article acceptance date: 16 March 2020

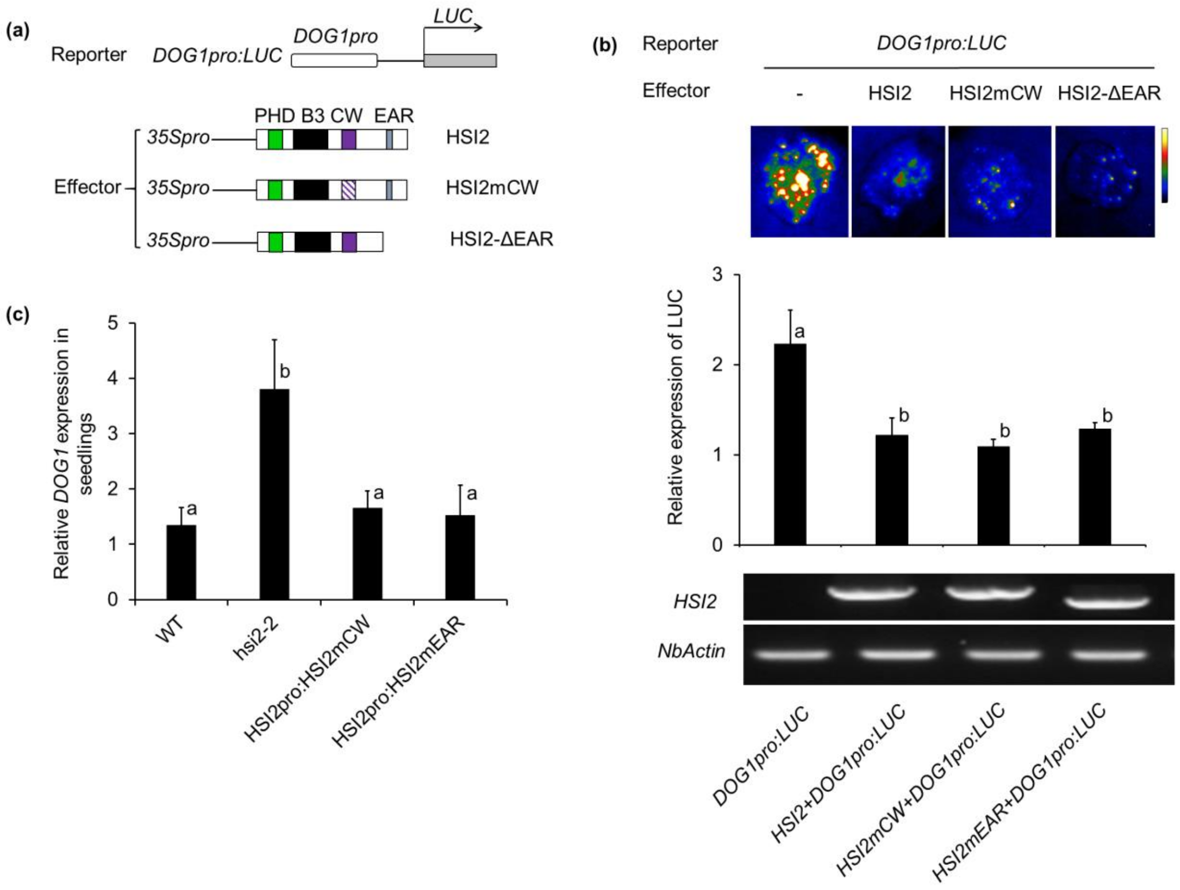

Figure S1

Fig. S1. Disruption of CW and EAR domains does not affect HSI2-mediated regulation of *DOG1* expression

(a) Schematic representation of reporter and effector used to test the function of the CW and EAR domains of HSI2. Effector constructs encodes either intact HSI2, HSI2mCW or HSI2-ΔEAR with an EAR motif deletion at the C-terminus of HSI2. **(b)** Luminescence images and relative expression analysis of the *LUC* mRNA, by RT-qPCR, from *N. benthamiana* leaves coinfiltrated with combinations of reporter and effector constructs, as indicated. RT-PCR analysis of *HSI2* and *NbActin* gene expression in infiltrated areas as above. **(c)** Relative expression of *DOG1* in Arabidopsis seedlings of WT, *HSI2pro:HSI2-HA, HSI2pro:HSI2mCW* and *HSI2pro:mEAR.* RT-qPCR assays were normalized using *EF1A*. Error bars indicate SD. Lowercase letters indicate significant differences ($P < 0.01$).

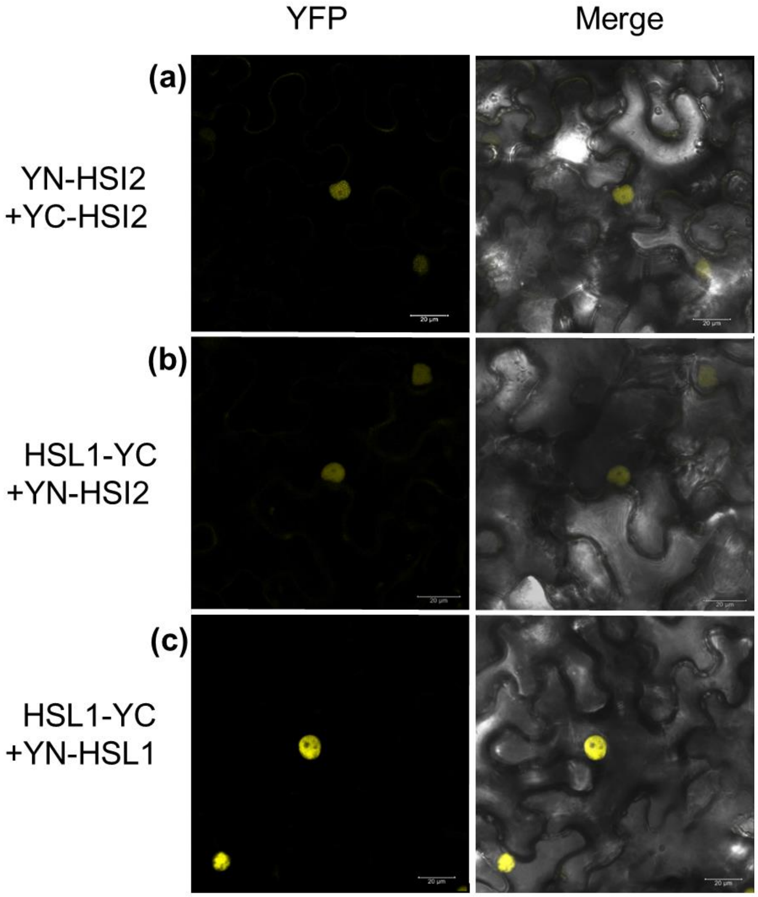

Figure S2

Fig. S2. HSI2 and HSL1 form homodimers and heterodimers *in vivo*.

BiFC analysis of protein-protein interactions between **(a)** HSI2 and HSI2, **(b)**, HSI2 and HSL1 and **(c)** HSL1 and HSL1.
